# Homoeolog expression bias and expression level dominance in resynthesized allopolyploid *Brassica napus*

**DOI:** 10.1186/s12864-018-4966-5

**Published:** 2018-08-06

**Authors:** Jian Wu, Li Lin, Meiling Xu, Peipei Chen, Dongxiao Liu, Qinfu Sun, Liping Ran, Youping Wang

**Affiliations:** Jiangsu Provincial Key Laboratory of Crop Genetics and Physiology, Yangzhou University, Yangzhou, 225009 China

**Keywords:** Oilseed rape, Allopolyploidization, Gene expression patterns, Homoeolog expression bias, Expression level dominance, RNA sequencing

## Abstract

**Background:**

Allopolyploids require rapid genetic and epigenetic modifications to reconcile two or more sets of divergent genomes. To better understand the fate of duplicate genes following genomic mergers and doubling during allopolyploid formation, in this study, we explored the global gene expression patterns in resynthesized allotetraploid *Brassica napus* (AACC) and its diploid parents *B. rapa* (AA) and *B. oleracea* (CC) using RNA sequencing of leaf transcriptomes.

**Results:**

We found that allopolyploid *B. napus* formation was accompanied by extensive changes (approximately one-third of the expressed genes) in the parental gene expression patterns (‘transcriptome shock’). Interestingly, the majority (85%) of differentially expressed genes (DEGs) were downregulated in the allotetraploid. Moreover, the homoeolog expression bias (relative contribution of homoeologs to the transcriptome) and expression level dominance (total expression level of both homoeologs) were thoroughly investigated by monitoring the expression of 23,766 *B. oleracea*-*B. rapa* orthologous gene pairs. Approximately 36.5% of the expressed gene pairs displayed expression bias with a slight preference toward the A-genome. In addition, 39.6, 4.9 and 9.0% of the expressed gene pairs exhibited expression level dominance (ELD), additivity expression and transgressive expression, respectively. The genome-wide ELD was also biased toward the A-genome in the resynthesized *B. napus*. To explain the ELD phenomenon, we compared the individual homoeolog expression levels relative to those of the diploid parents and found that ELD in the direction of the higher-expression parent can be explained by the downregulation of homoeologs from the dominant parent or upregulation of homoeologs from the nondominant parent; however, ELD in the direction of the lower-expression parent can be explained only by the downregulation of the nondominant parent or both homoeologs. Furthermore, Gene Ontology (GO) enrichment analysis suggested that the alteration in the gene expression patterns could be a prominent cause of the phenotypic variation between the newly formed *B. napus* and its parental species.

**Conclusions:**

Collectively, our data provide insight into the rapid repatterning of gene expression at the beginning of *Brassica* allopolyploidization and enhance our knowledge of allopolyploidization processes.

**Electronic supplementary material:**

The online version of this article (10.1186/s12864-018-4966-5) contains supplementary material, which is available to authorized users.

## Background

Polyploidy (often called whole-genome duplication) has been and continues to be a prominent and significant force in plant evolution [1, 2]. Most flowering plant lineages, even plants with relatively small genomes, such as *Arabidopsis thaliana,* are currently considered to reflect at least one round of ancient polyploidy in their evolutionary history [2, 3]. Two major categories of polyploidy exist in plants, i.e., autopolyploidy and allopolyploidy. Autopolyploidy contains multiple sets of the same or similar genomes derived from intraspecific genome duplication, whereas allopolyploidy combines two or more divergent homoeologous genomes derived from interspecific or intergeneric hybridization [4]. Allopolyploids exhibit an enhanced growth vigor and an advantage in ecological adaptation relative to autopolyploid and diploids and are thought to have played a significant role in plant diversification and speciation [5–7]. Many important crops are allopolyploids, including rapeseed (*Brassica napus*), wheat (*Triticum aestivum*) and cotton (*Gossypium hirsutum*).

After breaking down the hybridization barrier, newly formed allopolyploids undergo ‘genomic shock’ [8], which can lead to a myriad of genetic and epigenetic modifications. The genetic changes include epistasis, DNA loss, homoeologous recombination, gene conversion and ectopic recombination. Epigenetic changes, including DNA methylation, histone modification, transposon suppression/release and small RNA-mediated gene silencing, may occur at the transcriptional or posttranscriptional levels [4, 5, 7, 9–12]. The genetic and epigenetic changes in new allopolyploid genomes may lead to extensive gene expression [7, 10]. When two diverged genomes merge into a single cell, duplicate copies of genes with similar or redundant functions may alter their gene expression patterns, which takes several forms, including unequal parental contributions, transgressive upregulation or downregulation, silencing, and altered expression times and locations [4, 5, 13]. The alteration of gene expression patterns is a prominent cause of the phenotypic variation between newly formed allopolyploids and their parental species and may be the primary source of phenotypic novelty that may be selected and domesticated [4, 9].

Oilseed rape (*Brassica napus* L., AACC, 2n = 38) is the most important oleiferous *Brassica* crop and oil crop worldwide. Oilseed rape not only provides edible oil for human diets but also provides protein-rich feed for animals. Oilseed rape is a recently formed (~ 7500 years ago) allotetraploid originating from the natural hybridization of its two diploid progenitor species, *B. rapa* (AA, 2n = 20) and *B. oleracea* (CC, 2n = 18) [14, 15]. Resynthesized polyploids with known progenitors are excellent materials for dissecting gene expression and genomic changes during the early stages of allopolyploid formation. Hence, *B. napus*, which is an allotetraploid derived by hybridizing *B. rapa* and *B. oleracea*, provides an excellent opportunity for investigating the evolution of allopolyploids. To elucidate the genetic and epigenetic alterations that occur during the initial stage of *B. napus* formation, resynthesized *B. napus* has been extensively investigated. These studies have provided reports of chromosomal rearrangements [12, 16–20], chromosome pairings [21], transposon activation [22], epigenetic phenomena [20, 23, 24], gene expression changes [20, 25–30], protein expression changes [31, 32], and alternative splicing pattern changes [33]. However, knowledge regarding the extent and direction of ELD (total expression level of both homoeologs) and homoeolog expression bias (relative contribution of homoeologs to the transcriptome) changes in newly formed allotetraploid *B. napus* is limited.

Next-generation sequencing technologies enable researchers to study whole transcriptomes and offer greater power in distinguishing the expression of homologous genes [34]. Recently, the genomes of *B. napus* [15, 35, 36] and its two diploid progenitor species, *B. rapa* [37] and *B. oleracea* [38], were successfully sequenced. The released *Brassica* genome sequences and next-generation sequencing technologies provide an unprecedented opportunity to monitor duplicate gene expression modifications in resynthesized allopolyploid *B. napus*.

In this study, we conducted a transcriptomic analysis of resynthesized allotetraploid *B. napus* and its diploid parents to explore the gene expression modifications that occur during the initial stage of A- and C-genome mergers and duplication. In addition, we thoroughly investigated the homoeolog expression bias and ELD in newly synthesized allotetraploid *B. napus*. The results provide new insight into duplicate gene (homoeolog) expression alterations at the beginning of *Brassica* allopolyploidization and enhance our knowledge of allopolyploidization processes.

## Results

### Whole-genome resequencing of nascent resynthesized *B. napus* and its diploid parents

**Fig. 1:**
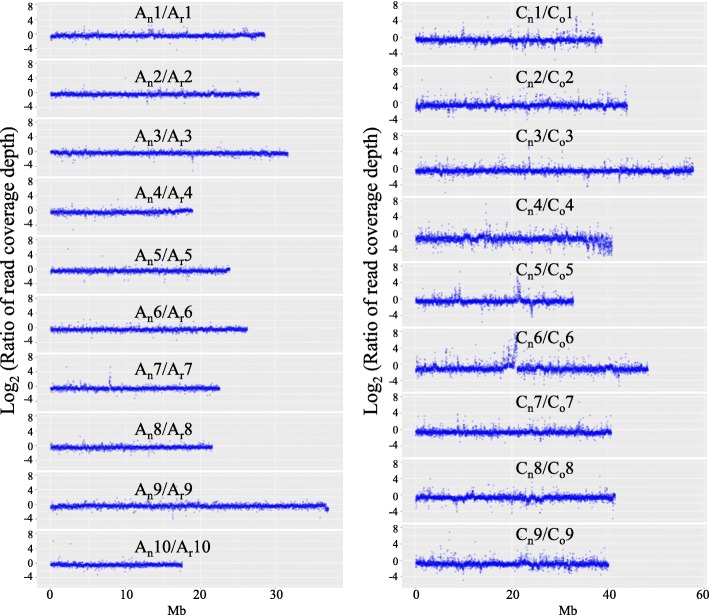
Whole-genome resequencing revealed that our newly resynthesized *B. napus* had a complete set of chromosomes. Coverage depth obtained for each chromosome after mapping the Illumina sequence reads from the resynthesized *B. napus* and its diploid parents to the reference genomes of *B. rapa* and *B. oleracea*. Each point represents a value in a 10-kb window

To confirm that our newly resynthesized *B. napus* had complete sets of chromosomes, we counted the chromosome numbers in root tip cells in our recent study [39]. Only the S0 plant, with 38 chromosomes, was used in the subsequent experiments. To further confirm that the newly resynthesized *B. napus* had a complete set of chromosomes, a resequencing analysis of the resynthesized *B. napus* (S1) and its diploid parents was performed. After mapping the Illumina sequence reads to the reference genomes, the coverage depth along each chromosome was obtained, and all 10 chromosomes in *B. rapa* and 9 chromosomes in *B. oleracea* were shown to be integrated (Fig. 1). Thus, our newly resynthesized *B. napus* had a complete set of 38 chromosomes.

### Transcriptome sequencing and read mapping

The RNA samples were isolated from the leaves of the resynthesized *B. napus* and its diploid parents at the 40-day-old seedling stage. Three biological replicates of each genotype were sampled. In total, 9 RNA libraries were subjected to paired-end RNA sequencing, and 443.4 million clean reads were obtained with an average of 49.3 million reads (7.2 Gb) in each sample (Table 1). On average, 72.5 and 75.2% of the reads from the ‘Yangzhouqing’ and ‘Yonglv 7’ samples were mapped to the *B. rapa* (A-genome) and *B. oleracea* (C-genome) genome sequences, respectively. Regarding the resynthesized *B. napus* samples, 72.2% (average of three biological replicates) of the reads were mapped to integrated genomes of the A- and C-genomes (Table 1). Of these mapped reads, approximately 86.0% were uniquely matched (Table 1).

**Table 1:** Statistics of RNA-seq reads and mapped reads

Samples^a^	Total raw reads	Total clean reads	Total clean nucleotides (bp)	Mapped reads (%)	Uniquely mapped reads (%)
AA-R1	63,235,082	62,967,506	8,973,601,893	76.7	68.4
AA-R2	69,562,114	68,900,748	10,037,616,256	75.6	68.2
AA-R3	32,752,136	30,728,480	4,609,272,000	65.3	60.0
CC-R1	50,069,024	49,125,050	7,075,517,555	80.3	73.1
CC-R2	69,523,248	68,890,164	9,995,606,828	81.3	46.8
CC-R3	36,039,320	32,996,098	4,949,414,700	64.1	59.1
AACC-R1	52,560,640	51,651,956	7,414,992,385	76.1	70.0
AACC -R2	48,235,332	47,798,278	6,801,312,765	77.5	60.0
AACC -R3	32,380,872	30,379,484	4,556,922,600	63.1	58.2
Total	454,357,768	443,437,764	64,414,256,982	–	–
Average	50,484,196	49,270,863	7,157,139,665	73.3	62.6

^a^R1, R2 and R3 are three biological replicates; AA, *B. rapa* (cv. Yangzhouqing); CC, *B. oleracea* (cv. Yonglv 7); AACC, resynthesized allotetraploid *B. napus*

**Fig. 2:**
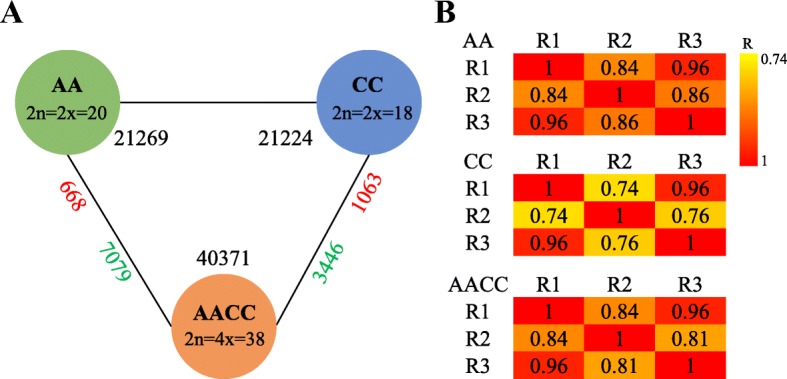
Transcriptome sequencing of nascent *B. napus* and its diploid parents. **a** DEGs between the progeny (AACC) and its diploid progenitors (AA and CC); the number of upregulated genes (red) is close to AA or CC, and the number of downregulated genes (blue) is near AACC. Numbers close to the species (black) represent the total number of expressed genes. **b** Pearson correlation coefficients between each pair of biological replicates under different sampling conditions. R1, R2 and R3 represent three biological replicates

The gene expression of the positively expressed genes was analyzed using an empirical cutoff value (FPKM ≥1) [5, 40]. In total, 21,269 genes expressed in ‘Yangzhouqing’ (AA) were detected; 21,224 and 40,371 genes were expressed in ‘Yonglv 7’ (CC) and resynthesized *B. napus*, respectively (Fig. 2a). Among the 40,371 genes expressed in *B. napus*, 20,136 genes were derived from the A-genome, and the remaining 20,235 genes were derived from the C-genome. The gene expression correlations between each pair of biological replicates were strong, with most Pearson correlation coefficients (*R*) > 0.81 (Fig. 2b). These results indicate that the sequencing data of the biological replicates were of high quality.

### DEGs in nascent resynthesized *B. napus*

To study the effects of allopolyploidization on gene expression in synthetic *B. napus*, we identified the DEGs between the synthetic *B. napus* (AACC) and its diploid parents (‘Yangzhouqing’, AA and ‘Yonglv 7’, CC). In total, 12,256 DEGs (30.4% of expressed genes) were identified. Among these DEGs, 7747 DEGs were present in the A-genome (AACC vs AA), while 4509 DEGs were present in the C-genome (AACC vs CC) (Fig. 2a). In both the A- and C-genomes of synthetic *B. napus*, most DEGs were downregulated relative to those in the diploid parents. In total, 91.4% (7079 of 7747) and 76.4% (3446 of 4509) of the DEGs in the A- and C-genomes were downregulated, respectively (Fig. 2a).

### Functional classifications of the DEGs

For the gene function annotation, all *B. rapa* and *B. oleracea* genes were initially searched against the Nr and InterPro databases, and 79,484 (92.9%) and 76,552 (89.5%) genes were annotated in these two databases, respectively. Then, GO annotation was performed by merging the Blast2GO and InterPro annotation results, and 72,143 (84.3%) genes were assigned to at least one GO term.

**Fig. 3:**
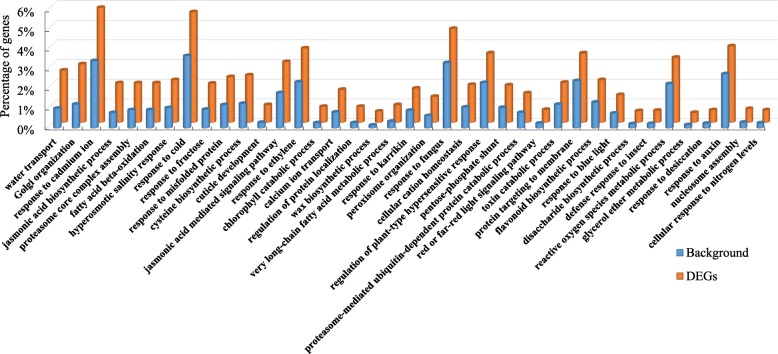
GO enrichment analysis of DEGs between resynthesized *B. napus* and its diploid parents. Only the biological process categorizations are shown. The y-axis represents the percentage of genes mapped by each term, representing the abundance of the GO terms. The percentage of the input list is calculated by the number of genes mapped to the GO term divided by the number of all genes in the input list. The same calculation was applied to the reference list (background) to generate its percentage

Then, we investigated the GO functional categories of all DEGs between the resynthesized *B. napus* and its diploid parents. In total, 57 significantly enriched GO terms were identified among all DEGs, including the following three categories: biological process (39 GO terms), cellular component (11 GO terms) and molecular function (7 GO terms) (Additional file 1). We focused on the 39 significantly enriched biological process terms and found that most enriched GO terms belonged to the following three secondary categories of biological processes: response to stimulus, metabolic process and cellular process (Fig. 3).

### Homoeolog expression bias in the resynthesized allotetraploid *B. napus*

Many studies have shown that duplicate gene pairs may display homoeolog expression bias in allotetraploids in which bias refers to the preferential expression of one homoeolog relative to the other [5, 13, 41, 42]. To study the homoeolog expression bias in the newly synthesized allotetraploid *B. napus*, we monitored the expression levels of the 23,766 *B. oleracea*-*B. rapa* orthologous gene pairs identified by Liu et al. [38].

**Fig. 4:**
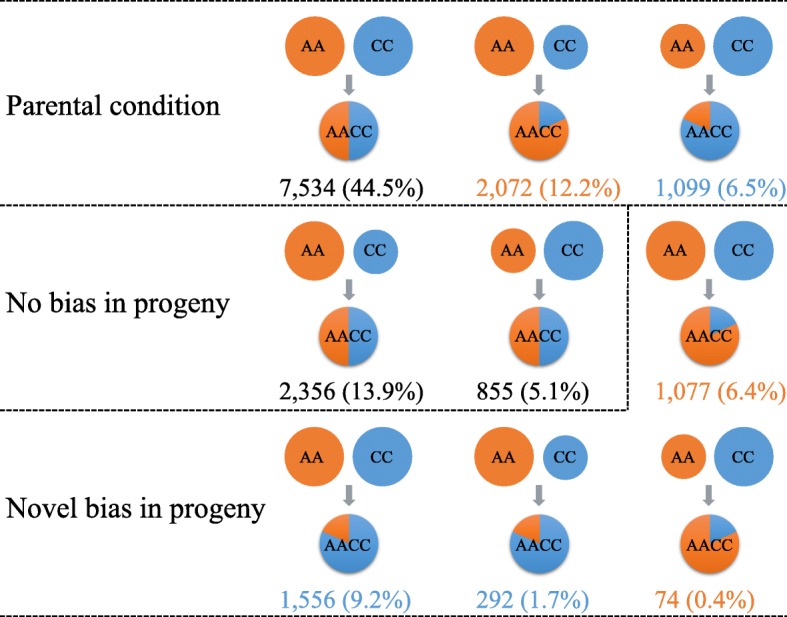
Homoeolog expression bias in resynthesized allotetraploid *B. napus*. The relative expression levels of the homoeologs are modeled by the size of the circles in the parental species *B. rapa* (AA) and *B. oleracea* (CC) or the area ratio of the circles in the progeny *B. napus* (AACC). The number of homoeolog pairs and its proportion to the total number of expressed homoeolog pairs are listed. In total, 3223 (19.1%) homoeolog pairs are A-biased in the progeny (orange numbers), 2947 (17.4%) homoeolog pairs are C-biases in the progeny (blue numbers), and 10,745 (63.5%) homoeolog pairs have no bias in the progeny (black numbers)

Of the 23,766 homoeolog pairs, 16,915 pairs were expressed (FPKM ≥1) in at least one of the three species. In total, 63.3% of all expressed homoeolog pairs (10,705 of 16,915 homoeolog pairs) in the resynthesized *B. napus* were maintained in the parental condition (Fig. 4). The expression patterns of more than half of the homoeolog genes in the diploid parents were conserved in the allopolyploid *B. napus*. Moreover, 17.7% (2999) of the homoeolog pairs displayed novel bias in the resynthesized *B. napus*, while 19.0% (3211) of the homoeolog pairs with preexisting expression bias in the parent reverted to nondifferential expression in the resynthesized *B. napus* (Fig. 4). Overall, 63.5% of the homoeolog pairs displayed no bias in the progeny resynthesized *B. napus*, and the remaining 36.5% showed biased expression (Fig. 4). Notably, the resynthesized allotetraploid showed unbalanced biased expression with a preference toward the A-genome (A-bias vs C-bias = 3223 (19.1%) vs 2947 (17.4%), Fig. 4).

### ELD in the resynthesized allotetraploid *B. napus*

In addition to expression bias, ELD has been frequently described recently. ELD does not consider the relative expression levels of individual homoeologs but rather refers to the total expression level of a homologous gene pair in an allopolyploid compared with the relative expression levels in its two parents [5, 13, 41, 43, 44]. To detect additivity, transgressive expression and expression level dominance (ELD) in the newly synthesized *B. napus*, we classified the expressed homoeolog pairs into 12 categories by comparing the total expression of the homoeologs in the allotetraploid relative to the expression observed in the diploid parents, as described by Yoo et al. [13].

**Fig. 5:**
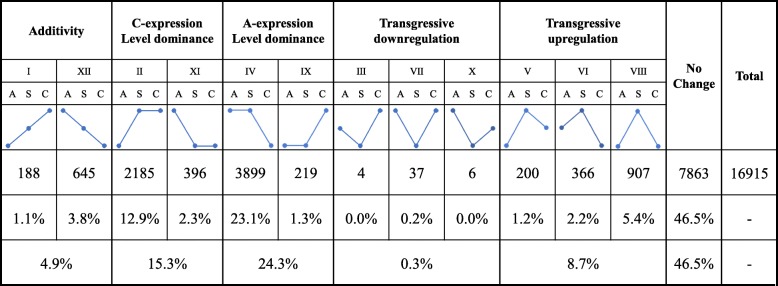
ELD in the resynthesized *B. napus*. The ELD is evaluated by comparing the total expression of the homoeologs in the allotetraploid (S) relative to the expression levels found in the parental species *B. rapa* (A) and *B. oleracea* (C)

Overall, 46.5% (7863 of 16,915) of the homoeolog pairs displayed equivalent expression (total expression level of a homologous gene pair equal to that in both diploid parents, ‘no change’ in Fig. 5) in the synthesized *B. napus*. More than 6699 (39.6%) homoeolog pairs showed ELD (categories II, XI, IV and IX, Fig. 5). Significantly more A-expression level dominance gene pairs (ELD-A, categories IV and IX, 24.3%) were observed than C-expression level dominance gene pairs (ELD-C, categories II and XI 15.3%, Fig. 5). Thus, the gene expression in the nascent synthesized *B. napus* displayed ELD bias toward the A-genome (*B. rapa*). Only 833 (4.9%, categories I and XII) gene pairs exhibited additivity expression. Moreover, 1520 (9.0%) gene pairs displayed transgressive expression. Notably, nearly all transgressive regulation gene pairs were transgressive upregulation (8.7% transgressive upregulation vs 0.3% transgressive downregulation, Fig. 5). Thus, most homologous gene pairs displayed dominant and equivalent expression in the newly synthesized *B. napus*, which may be due to duplicate copies of genes with similar or redundant functions altering their gene expression patterns during the initial stage of the A- and C-genome merger.

### Relationship between individual homoeolog expression and ELD

**Fig. 6:**
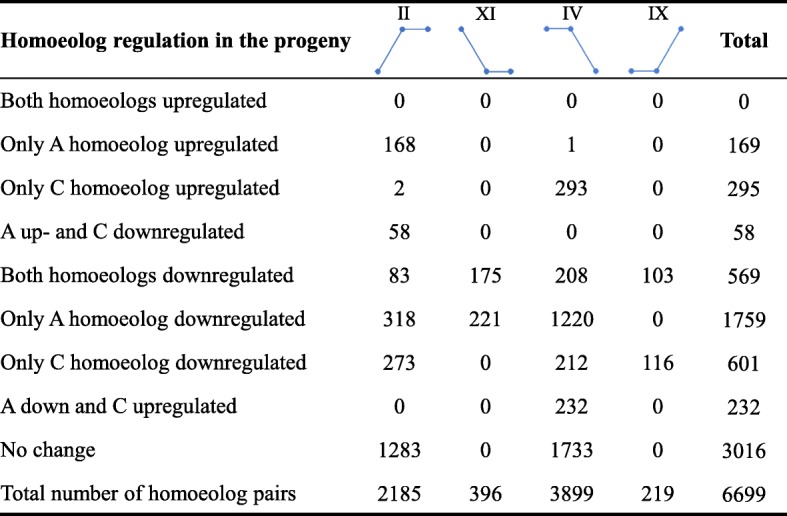
Homoeolog expression levels relative to the levels of its diploid parents explained the phenomenon of ELD. The figures shown at the top are parallel to those shown in Fig. 5 and represent the 4 differential expression states of ELD

To explain the phenomenon of ELD, we investigated the individual homoeolog expression levels relative to those of the diploid parents. In up to 55% ((6699–3016)/6699) of the ELD-A and ELD-C homoeolog pairs, the main reason for the ELD was that either one or both of the homoeologs modified their expression after the A- and C-genome merger (Fig. 6). Most homoeolog expression modifications were downregulations, reflecting the downregulation of the alternative homoeolog (2360 pairs = 1759 + 601) or both homoeologs (569 pairs). In addition, we observed more modifications in the A homoeolog of the gene pairs (2787 genes = 169 + 58 + 569 + 1759 + 232) than in the C homoeolog (1755 genes = 295 + 58 + 569 + 601 + 232) (Fig. 6). For the gene pairs in ELD-A category IV and ELD-C category II, the dominant parent had a higher expression than the nondominant parent (ELD higher-expression parent). This ELD can mostly be explained by the downregulation of the homoeolog from the dominant parent (1660 (208 + 1220 + 232) pairs in IV, and 414 (58 + 83 + 273) pairs in II) or the upregulation of the homoeolog from the nondominant parent (525 (293 + 232) pairs in IV, and 226 (168 + 58) pairs in II) (Fig. 6). For the gene pairs in ELD-A category IX and ELD-C category XI (ELD lower-expression parent), the dominant parent had a lower expression than did the nondominant parent (Fig. 6). Here, ELD can be explained only by the downregulation of the homoeolog from the nondominant parent (116 pairs in IX, and 221 pairs in XI) or downregulation of both homoeologs (103 pairs in IX, and 175 pairs in XI).

### Functions of genes exhibiting ELD

We further investigated the possible functions of the genes that exhibited ELD according to the GO terms of biological process and found that the ELD-A genes were enriched with distinct GO terms compared with the ELD-C genes (Table 2, Additional file 2). Four of the top 5 significantly enriched biological process terms for the ELD-A genes belong to responses to stimuli, such as regulation of plant-type hypersensitive response, negative regulation of programmed cell death, response to chitin, and response to cadmium ion. By contrast, three of the top 5 significantly enriched biological process terms for the ELD-C genes belong to metabolic processes, such as gluconeogenesis, isopentenyl diphosphate biosynthetic process, methylerythritol 4-phosphate pathway and photorespiration. Interestingly, the ELD-A genes were enriched with GO terms associated with pigment biosynthetic and metabolic processes, such as carotenoid biosynthetic process, carotene biosynthetic process, chlorophyll catabolic process and anthocyanin accumulation in tissues in response to UV light (Additional file 2), which may partially explain why the colors of the leaves, petals and seed coats of our resynthesized *B. napus* predominantly resembled the parent *B. rapa* [39].

**Table 2:** GO enrichment analysis of expression-level dominance genes in the resynthesized *B. napus*

Expression patterns	GO ID^a^	GO Name	FDR
ELD-A	GO:0006612	protein targeting to membrane	5.49E-20
	GO:0010363	regulation of plant-type hypersensitive response	5.77E-19
	GO:0043069	negative regulation of programmed cell death	1.79E-18
	GO:0010200	response to chitin	2.91E-17
	GO:0046686	response to cadmium ion	8.27E-17
ELD-C	GO:0046686	response to cadmium ion	1.86E-21
	GO:0006626	protein targeting to mitochondrion	3.72E-21
	GO:0006094	gluconeogenesis	1.44E-13
	GO:0019288	isopentenyl diphosphate biosynthetic process, methylerythritol 4-phosphate pathway	9.97E-13
	GO:0009853	photorespiration	7.10E-10
Transgressive up-regulation	GO:0046686	response to cadmium ion	2.49E-24
	GO:0006096	glycolytic process	8.55E-18
	GO:0006094	gluconeogenesis	8.99E-18
	GO:0009409	response to cold	3.72E-17
	GO:0009744	response to sucrose	7.62E-15
Transgressive down-regulation	–	–	–
Additivity	GO:0006499	N-terminal protein myristoylation	2.02E-09
	GO:0015996	chlorophyll catabolic process	5.78E-07
	GO:0080086	stamen filament development	1.66E-06
	GO:0009651	response to salt stress	1.75E-05
	GO:0042398	cellular modified amino acid biosynthetic process	2.33E-05

^a^The top 5 significantly enriched biological process terms for each type of expression patterns and all significantly enriched GO terms are listed in Additional file 2

### Validation of RNA-seq analysis by qRT-PCR

**Fig. 7:**
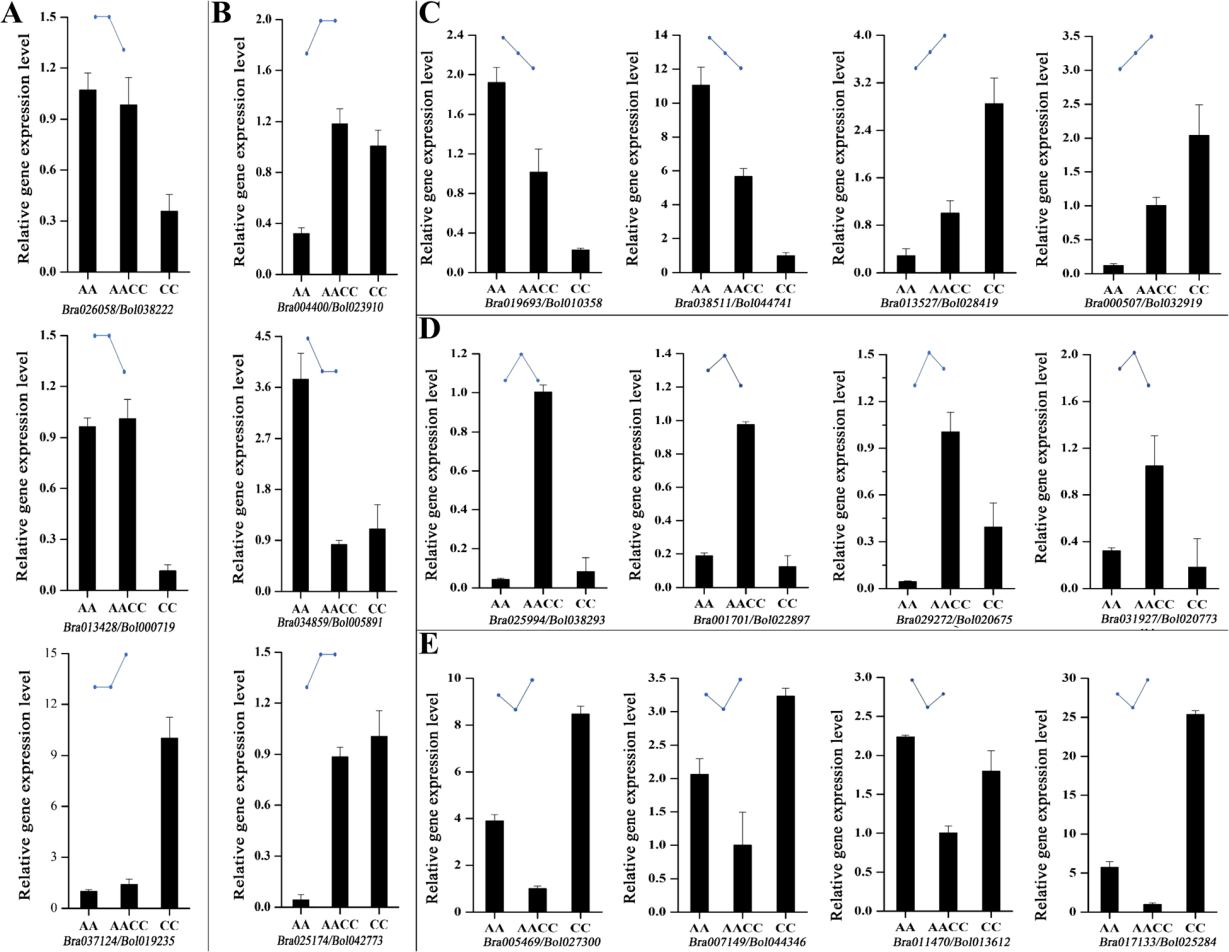
qRT-PCR confirmation of ELD in the resynthesized *B. napus*. Error bars represent the standard deviation from three biological replicates. The expression patterns of each categories derived from the RNA-seq data are shown above each histogram. **a** A-ELD; **b** C-ELD; **c** additivity; **d** transgressive upregulation; **e** transgressive upregulation

To validate the data obtained by RNA-seq, 18 orthologous gene pairs were subjected to qPCR assays. Three or four gene pairs representing additivity, ELD-C, ELD-A, transgressive downregulation and transgressive upregulation were selected (Fig. 5). The relative expression levels in the synthesized *B. napus* and its parents were compared with those in the RNA-seq data (FPKM value). For all 18 orthologous gene pairs, the qRT-PCR analysis revealed the same expression patterns as the RNA-seq data (Fig. 7), demonstrating the reliability of the data produced by RNA-seq.

## Discussion

How allopolyploidy reconciles two or more sets of diverged genomes and regulatory interactions is a tantalizing question. *B. napus* was formed by recent allopolyploidy (~ 7500 years ago) between ancestors of *B. oleracea* and *B. rapa* [15] and serves as a model system for investigations of the consequences of hybridization and genome duplication on the allopolyploid genome [23]. The genome sequencing of *B. napus* showed that incipient gene loss and expression divergence have likely begun since its formation [15]. Hence, in the present study, we employed a resynthesized *B. napus* line that was generated by interspecific hybridization between two known diploid parents, *B. rapa* (‘Yangzhouqing’) and *B. oleracea* (‘Yonglv 7’), to investigate the early consequences of allopolyploid formation on gene expression. Specifically, homoeolog expression bias and ELD in the resynthesized allopolyploid *B. napus* were investigated in this study.

### Transcriptomic shock during *B. napus* allopolyploidization

Previously, a 70-mer oligo microarray containing 26,107 annotated *Arabidopsis* genes was used to analyze the gene expression in resynthesized *B. napus* [28]. Due to the advent of next-generation sequencing technologies, whole transcriptome changes in resynthesized *Brassica* allotetraploids have been investigated using 35 bp [25] or 80 bp [26] single-end sequencing. However, due to whole-genome duplication, distinguishing the expression patterns of homologous genes in *B. napus* by *Arabidopsis*-specific microarray or short single-end reads is challenging. In the current study, we employed 2 × 151 bp paired-end sequencing, which has much greater power in distinguishing between homoeologous genes and studying whole transcriptomes. On average, approximately 86.0% of all mapped reads in all samples could be unambiguously mapped to unique regions of the A- and C- genomes (Table 1). Only the uniquely mapped reads were considered in the gene expression analysis. In addition, previous studies have only used the *B. rapa* reference genome for all three species (*B. rapa*, *B. oleracea* and resynthesized *B. napus*) [25, 26], making it difficult to distinguish A and C homoeologs in resynthesized *B. napus*. In this study, we used *B. rapa*, *B. oleracea* and integrated genome sequences as the reference genomes for ‘Yangzhouqing’, ‘Yonglv 7’ and the resynthesized *B. napus*, respectively. Given these advantages, the transcript abundances of homoeologous genes can be accurately measured.

In this study, we found that approximately one-third (12,256/40,371) of the expressed genes were differentially expressed between the resynthesized *B. napus* and its diploid parents (Fig. 2a), suggesting that extensive changes in the patterns of parental gene expression occurred during the initial stage of *B. napus* formation. This phenomenon has been termed ‘transcriptomic shock’ [45, 46] and is commonly observed in allopolyploids, such as common wheat [5], cotton [13] and *Senecio* [46]. Strikingly, most (85%) DEGs between the resynthesized *B. napus* and its diploid parents were downregulated. However, the result is inconsistent with that reported by Jiang et al. [25] and Zhang et al. [26]. Jiang et al. [25] drew a completely opposite conclusion, determining that 87.5% of the DEGs were upregulated in resynthesized *B. napus*. Zhang et al. [26] found that the numbers of upregulated and downregulated DEGs were almost equal in resynthesized *B. napus*. When two diverged genomes merge into a single cell, the increased gene or genome dosage may induce disease syndromes and abnormal development; thus, the expression of orthologous genes with similar or redundant functions must be reprogrammed [7]. The reprogramming of gene expression in our study constituted a downregulation of most DEGs during the early process of *Brassica* allopolyploidization.

### Approximately one-third of the expressed gene pairs displayed expression bias with a slight preference toward the A-genome in resynthesized *B. napus*

Previous studies have shown that certain duplicate genes may diverge expression patterns in response to the consequences of genome duplication [9, 13, 43]. In the present study, we use the terminologies ‘homoeolog expression bias’ and ‘ELD’ to describe the alterations in the duplicate gene expression patterns in the resynthesized allopolyploids *B. napus*.

The term ‘homoeolog expression bias’ refers to the preferential expression of one homoeolog relative to the other in resynthesized *B. napus* (A and C homoeologs contribute unequally to the total gene expression). This phenomenon has been documented in many different allopolyploids, particularly in *Triticum* [5, 47, 48] and *Gossypium* [13, 49, 50]. In this study, we found that approximately 36.5% of the 16,915 expressed homoeolog pairs displayed expression bias (Fig. 4). A similar proportion of significantly biased genes were found in the *B. napus* cultivar ‘Darmor-bzh’ (~ 33% of the gene pairs in both the leaves and roots) [15]. The proportion of significantly biased genes in *B. napus* was slightly higher than that in synthetic allopolyploid cotton (25.5%) [13] and synthetic hexaploid wheat (27.0%) [5].

Specifically, slightly more genes exhibited expression bias toward the A homoeologs (19.1%) than toward the C homoeologs (17.4%) (Fig. 4). However, more gene pairs showed C-biased expression (17.3%) than A-biased expression (15.7%) in the naturally cultivated ‘Darmor-bzh’ [15]. A similar result was found in *B. juncea*; in all-natural *B. juncea*, homoeolog expression biased genes were derived predominantly from the B-genome, whereas one of the two transcriptomes derived from the resynthesized *B. juncea* types showed expression bias toward the A-genome [51]. These inconsistent results between natural and resynthesized *Brassica* allotetraploids may be because the domestication process affected the expression patterns of duplicate genes.

### Gene expression in nascent resynthesized *B. napus* displayed ELD bias toward the A-genome

The term ‘ELD’ describes the phenomenon in which the total expression level of a homologous gene pair in resynthesized *B. napus* is statistically the same as that in only one of the two diploid parents. This term was initially proposed by Grover et al. [41] and previously called ‘genomic dominance’ [43]. In this study, we classified the expressed homoeolog pairs into 12 categories by comparing the total expression of the homoeologs in the resynthesized *B. napus* relative to the expression levels found in the parental species following the method applied by Yoo et al. [13] in cotton.

We found that 39.6% (6699 pairs) of the 16,915 expressed homoeolog pairs showed ELD in resynthesized *B. napus* (Fig. 5). Interestingly, more ELD-A (24.3%) than ELD-C gene pairs (15.3%) were found in the resynthesized *B. napus* (Fig. 5), suggesting that genes in the nascent resynthesized *B. napus* displayed ELD bias toward the A-genome. In addition, we investigated how homoeolog expression contributes to ELD and found that ELD in the direction of the higher-expression parent can be explained by the downregulation of the homoeolog from the dominant parent or the upregulation of the homoeolog from the nondominant parent; however, the ELD in the direction of the lower-expression parent can be explained only by the downregulation of the nondominant parent or both homoeologs (Fig. 6).

Previous studies have suggested a hierarchy of nucleolar dominance in three *Brassica* allotetraploids (genomes BB > AA > CC) in which both *B. juncea* (BB > AA) and *B. carinata* (BB > CC) expressed rRNA genes from the B-genome, and *B. napus* (AA > CC) expressed rRNA genes from the A-genome [52–54]. A recent study revealed that the distinct subgenome stability was BB > AA > CC in synthesized *Brassica* allohexaploids (2n = 54, AABBCC) [55]. Hence, both nucleolar dominance and subgenome stability were AA > CC. These findings may partially explain our results in which both homoeolog expression bias and ELD showed preference toward the A-genome in resynthesized *B. napus*.

In a recent study, *B. napus* hybrids (2n = 19, AC) between the restituted and extant *B. rapa* and the same *B. oleracea* genotype were studied via RNA-seq and compared with a natural *B. napus* donor to reveal the gene expression changes duo to hybridization and domestication [30]. However, the results indicated that expression level dominance and homoeolog expression bias were balanced at the initial stage of genome merger, which is inconsistent with our results. The inconsistent results may have occurred because both genome merger and genome doubling altered the transcriptome in our study, while only genome merger altered the transcriptome in the previous study.

### Different phenotypes and enhanced growth vigor of *B. napus* may partially be attributed to ELD

The GO enrichment analysis showed that, compared with the ELD-C genes, the proposed ELD-A genes were enriched with distinct GO terms (Table 2, Additional file 2). More interestingly, our resynthesized *B. napus* predominantly resembled the parent *B. rapa* in certain phenotypes, such as leaf color, petal color, and seed coat color [39]. These data may have occurred because the ELD-A genes were enriched with GO terms associated with pigment biosynthetic and metabolic processes (Additional file 2).

Furthermore, the allotetraploid *B. napus* had different phenotypes compared with its diploid parents, particularly in terms of hybrid vigor, such as more robust seedling growth, higher plant height, more branches, more leaves, larger flower size and thicker siliques [39]. In addition, certain GO terms associated with carbohydrate catabolic and biosynthetic processes, such as the ‘glycolytic process’, ‘gluconeogenesis’, ‘maltose metabolic process’, ‘starch biosynthetic process’ and the ‘xyloglucan biosynthetic process’, were significantly enriched in transgressive upregulated genes (Table 2, Additional file 2), which may contribute to the growth vigor of the resynthesized *B. napus* and explain its higher potential seed yield, leading *B. napus* to become the most important oleiferous *Brassica* crop worldwide.

## Conclusions

In conclusion, the present study employed a synthetic *B. napus* allotetraploid and its diploid parents, *B. rapa* and *B. oleracea*, to investigate the fate of duplicated genes following genomic mergers and doubling during allopolyploid formation. Our results suggested that newly formed *B. napus* undergo ‘transcriptomic shock’. Approximately one-third of the expressed gene pairs displayed expression bias with a slight preference toward the A-genome. Moreover, 39.6% of the expressed gene pairs exhibited ELD and were biased toward the A-genome. We propose that the alteration of gene expression patterns could be a prominent cause of the phenotypic variation observed between the newly formed *B. napus* and its parental species. The results provide new insight into changes in duplicate gene expression at the beginning of *Brassica* allopolyploidization.

## Methods

### Plant material and sample preparations

*B. rapa* cv. ‘Yangzhouqing’ (AA, 2*n* = 20) and *B. oleracea* cv. ‘Yonglv 7’ (CC, 2*n* = 18) were obtained from the Jiangsu Lixiahe Region Agricultural Research Institute, China. ‘Yangzhouqing’ is a cultivar with good cold tolerance and resistance to soft rot, making it popular in the Yangtze River Delta of China. ‘Yonglv 7’ belongs to the *B. oleracea* Italica group, and has been used as a parent germplasm for broccoli breeding. A crossing experiment was conducted in the field of Yangzhou University by using *B. oleracea* as the pollen donor and *B. rapa* as the seed parent, as described in our recent study [39]. Siliques were collected 7 days after pollination (DAP) and sterilized before embryo rescue on MS medium (containing 500 mg/L casein hydrolysate and 3% sucrose). After 35 days, all the seeds were stripped and cultivated on MS medium for hybrid regeneration. Chromosome doubling using 0.2% colchicine was carried at the four-leaf stage. S_1_ seeds were obtained from diploid F_1_ hybrids (AC) after colchicine-induced chromosome doubling (S_0_) and were grown as the second generation of allotetraploid plants (AACC). All of the resynthesized *B. napus* (S_1_) plants and plants of its two diploid parents were grown in the experimental field of Yangzhou University, Yangzhou, China. The fourth true leaves from four plants of each genotype at the same physiologic stage (40-day-old seedlings) were pooled. Three biological replicates were performed. The harvested tissues were immediately frozen in liquid nitrogen and stored at − 80 °C.

### RNA extraction, cDNA library construction and RNA sequencing

Total RNA was extracted using the TRIzol reagent (Invitrogen, Carlsbad, CA) following the manufacturer’s instructions, and treated with RNase-free DNase I (Thermo Scientific, USA) to remove any contaminating genomic DNA. In total, 9 RNA samples (3 samples of each genotype) were subjected to library construction using an Illumina® TruSeq™ RNA Sample Preparation Kit, following the manufacturer’s instructions. Then, all mRNA-seq libraries were sequenced using an Illumina HiSeq 3000 sequencer at the National Key Laboratory of Crop Genetic Improvement, Huazhong Agricultural University. Sequencing of paired-end reads of 2 × 151 bp in length was performed. The original data set was deposited in the NCBI Sequence Read Archive (accession No. SRP139144).

### Alignment of RNA-seq reads to reference genomes

Various quality controls for the raw reads were conducted using a next-generation sequencing (NGS) QC tool kit [56] to (i) remove reads containing primer/adaptor sequences and low-quality reads [number of bases with PHRED-like scores (Q-scores) less than 20 exceeding 30%], (ii) trim the low-quality bases (Q-score < 20) from the 3′ ends of the reads, and (iii) remove reads that were less than 80 bp in length.

All high-quality reads from each sample that passed the quality control assay were aligned to the reference genomes with HISAT2 v2.0.4 using the default parameters [57]. *B. rapa* cv. ‘Chiifu-401-42’ genome v1.5 (A-genome) [37] and *B. oleracea* cv. ‘capitata line 02-12’ genome v1.0 (C-genome) [38] were used as the reference genomes for ‘Yangzhouqing’ and ‘Yonglv 7’, respectively. The A- and C-genomes were integrated and served as the reference genomes for the resynthesized *B. napus*. Only uniquely mapped reads were considered in the analysis. Differential gene expression and transcript abundance were calculated using the Cufflinks v2.2.1 program [58]. The transcript abundance of each gene was estimated based on the fragments per kilobase of transcript per million fragments mapped (FPKM) value. Genes with a false discovery rate (FDR) ≤ 0.05 and an absolute log2-fold change value ≥1 between the resynthesized *B. napus* and its two parents were defined as differentially expressed genes (DEGs).

### Gene ontology enrichment analysis

The Gene Ontology (GO) terms for the entire set of *B. rapa* and *B. oleracea* genes were annotated as described by Wu et al. [40]. First, all genes were searched against the NCBI non-redundant (Nr) protein database using BlastP with an E-value ≤1E-05 and the InterPro database (http://www.ebi.ac.uk/interpro/) using InterProScan5 [59]. Then, the GO terms associated with each BLAST hit were annotated using Blast2GO [60]. Finally, the GO terms of each gene were annotated by merging the Blast2GO and InterPro annotation results. GO enrichment analyses were performed using Blast2GO, and GO terms with an FDR ≤ 0.001 were considered significantly enriched. REVIGO was employed to reduce redundancies in the significantly enriched GO terms using a similarity cutoff value = 0.75 [61].

### Analyses of ELD and homoeolog expression bias

To study the changes in ELD (total expression levels of both homoeologs) and homoeolog expression bias (relative contribution of homoeologs to the transcriptome) in the newly synthesized allotetraploid *B. napus*, we monitored the expression levels of the 23,766 *B. oleracea*-*B. rapa* orthologous gene pairs identified by Liu et al. [38]. Sequence differences between any pair of orthologous genes allowed us to distinguish homoeolog expression according to the homoeolog-specific reads. In the analysis of homoeolog expression bias analysis, we compared the expression level of each homoeolog pair in the diploid parents (A_r_ vs C_o_) and the synthesized *B. napus* (A_n_ vs C_n_) using Student’s *t*-test (*P* ≤ 0.05). In the ELD analysis, we compared the total expression level of a homologous gene pair in the synthesized *B. napus* to that in the diploid parents (i.e., A_n_ + C_n_ vs A_r_ and A_n_ + C_n_ vs C_o_) using Student’s *t*-test (*P* ≤ 0.05). Twelve possible classes of differential expression (see Fig. 5), including ELD, additivity and transgressive (outside the range of either parent), were classified according to Yoo et al. [13].

### Whole-genome resequencing of the resynthesized *B. napus* and its diploid parents

Genomic DNA was extracted from the young leaves (the same samples used for RNA extraction) of the resynthesized *B. napus* and its diploid parents following the CTAB DNA extraction procedure. DNA libraries with an insert size of approximately 500 bp were constructed following the manufacturer’s instructions (Illumina® TruSeq™ RNA Sample Preparation Kit), and 2 × 151 bp paired-end reads were generated using an Illumina HiSeq 3000 sequencer at the National Key Laboratory of Crop Genetic Improvement, Huazhong Agricultural University.

Various quality controls procedures were performed as described in the RNA-seq section above. All high-quality reads in each sample were aligned to the reference genomes with Bowtie v2.2.5 using the default parameters [62]. Only uniquely mapped reads were employed for coverage depth calling. The depth at each base pair was attained by calling SAMtools depth [63]. We calculated the ratio in a 10-kb window for each chromosome as follows to estimate chromosomal integrity: (coverage depth on A_n_) / (coverage depth on A_r_) and (coverage depth on C_n_) / (coverage depth on C_o_).

### qRT-PCR validation

Thirty-six DEGs (18 homoeolog gene pairs) were chosen for validation of the RNA-seq data using qRT-PCR. The RNA samples employed for qRT-PCR analysis were the same as those employed for the RNA-seq experiments. First-strand cDNA was synthesized from 2 μg of total RNA from each sample using the RevertAid™ First Strand cDNA Kit (Fermentas, Thermo Fisher Scientific, Waltham, MA, USA) according to the described protocol. Homoeolog gene pair specific primers were designed using Primer 5.0 software, and all primer sequences are listed in Additional file 3. The reactions were conducted using SYBR Green PCR Master Mix in a Stratagene Mx3005P Quantitative PCR instrument (Agilent, USA). The thermal cycling program was set to 50 °C for 2 min; 95 °C for 1 min; and 40 cycles of 95 °C for 15 s and 60 °C for 45 s. The relative expression data were derived from three technical replicates of each sample. The *Actin2* gene was used as an internal control for data normalization, and the relative expression level was calculated using the delta-delta threshold cycle (Ct) method.

## Additional files

Additional file 1: GO enrichment analysis of DEGs between resynthesized *B. napus* and its diploid parents. Three major functional categories were grouped: biological process (P), cellular component (C) and molecular function (F). (XLSX 10 kb)

Additional file 2: GO enrichment analysis of ELD genes in the resynthesized *B. napus*. (XLSX 12 kb)

Additional file 3: Primer sequences used for qRT-PCR analyses. (XLSX 19 kb)

## Abbreviations

DEGs: Differentially expressed genes
ELD: Expression level dominance
FDR: False discovery rate
FPKM: Fragments per kilobase of transcript per million fragments mapped
GO: Gene Ontology

## References

[1] Adams KL, Wendel JF (2005). Polyploidy and genome evolution in plants. Curr Opin Plant Biol.

[2] Jiao Y, Wickett N, Ayyampalayam S, Chanderbali A, Landherr L, Ralph P, Tomsho L, Hu Y, Liang H, Soltis P (2011). Ancestral polyploidy in seed plants and angiosperms. Nature.

[3] Vision TJ, Brown DG, Tanksley SD (2000). The origins of genomic duplications in *Arabidopsis*. Science.

[4] Doyle JJ, Flagel LE, Paterson AH, Rapp RA, Soltis DE, Soltis PS, Wendel JF (2008). Evolutionary genetics of genome merger and doubling in plants. Annu Rev Genet.

[5] Li A, Liu D, Wu J, Zhao X, Hao M, Geng S, Yan J, Jiang X, Zhang L, Wu J (2014). mRNA and small RNA transcriptomes reveal insights into dynamic homoeolog regulation of allopolyploid heterosis in nascent hexaploid wheat. Plant Cell.

[6] Abbott R, Albach D, Ansell S, Arntzen JW, Baird SJ, Bierne N, Boughman J, Brelsford A, Buerkle CA, Buggs R (2013). Hybridization and speciation. J Evolution Biol.

[7] Chen ZJ (2007). Genetic and epigenetic mechanisms for gene expression and phenotypic variation in plant polyploids. Annu Rev Plant Biol.

[8] McClintock B (1984). The significance of responses of the genome to challenge. Science.

[9] Jackson S, Chen ZJ (2010). Genomic and expression plasticity of polyploidy. Curr Opin Plant Biol.

[10] Springer NM, Lisch D, Li Q (2016). Creating order from chaos: epigenome dynamics in plants with complex genomes. Plant Cell.

[11] Ha M, Lu J, Tian L, Ramachandran V, Kasschau KD, Chapman EJ, Carrington JC, Chen X, Wang X-J, Chen ZJ (2009). Small RNAs serve as a genetic buffer against genomic shock in *Arabidopsis* interspecific hybrids and allopolyploids. P Natl Acad Sci USA..

[12] Xiong Z, Gaeta RT, Pires JC (2011). Homoeologous shuffling and chromosome compensation maintain genome balance in resynthesized allopolyploid *Brassica napus*. P Natl Acad Sci USA..

[13] Yoo M, Szadkowski E, Wendel J (2013). Homoeolog expression bias and expression level dominance in allopolyploid cotton. Heredity.

[14] Nagaharu U (1935). Genome analysis in *Brassica* with special reference to the experimental formation of *B. napus* and peculiar mode of fertilization. Jpn J Bot.

[15] Chalhoub B, Denoeud F, Liu S, Parkin IA, Tang H, Wang X, Chiquet J, Belcram H, Tong C, Samans B (2014). Early allopolyploid evolution in the post-Neolithic *Brassica napus* oilseed genome. Science.

[16] Song K, Lu P, Tang K, Osborn TC (1995). Rapid genome change in synthetic polyploids of *Brassica* and its implications for polyploid evolution. P Natl Acad Sci USA..

[17] Gaeta RT, Pires JC, Iniguez-Luy F, Leon E, Osborn TC (2007). Genomic changes in resynthesized *Brassica napus* and their effect on gene expression and phenotype. Plant Cell.

[18] Szadkowski E, Eber F, Huteau V, Lodé M, Huneau C, Belcram H, Coriton O, Manzanares-Dauleux M, Delourme R, King GJ (2010). The first meiosis of resynthesized *Brassica napus*, a genome blender. New Phytol.

[19] Szadkowski E, Eber F, Huteau V, Lodé M, Coriton O, Jenczewski E, Chevre A (2011). Polyploid formation pathways have an impact on genetic rearrangements in resynthesized *Brassica napus*. New Phytol.

[20] Cui C, Ge X, Zhou Y, Li M, Li Z (2013). Cytoplasmic and genomic effects on non-meiosis-driven genetic changes in *Brassica* hybrids and allotetraploids from pairwise crosses of three cultivated diploids. PLoS One.

[21] Cui C, Ge X, Gautam M, Kang L, Li Z (2012). Cytoplasmic and genomic effects on meiotic pairing in *Brassica* hybrids and allotetraploids from pair crosses of three cultivated diploids. Genetics.

[22] Sarilar V, Palacios PM, Rousselet A, Ridel C, Falque M, Eber F, Chèvre AM, Joets J, Brabant P, Alix K (2013). Allopolyploidy has a moderate impact on restructuring at three contrasting transposable element insertion sites in resynthesized *Brassica napus* allotetraploids. New Phytol.

[23] Xu Y, Zhong L, Wu X, Fang X, Wang J (2009). Rapid alterations of gene expression and cytosine methylation in newly synthesized *Brassica napus* allopolyploids. Planta.

[24] Ran L, Fang T, Rong H, Jiang J, Fang Y, Wang Y (2016). Analysis of cytosine methylation in early generations of resynthesized *Brassica napus*. J Integr Agr.

[25] Jiang J, Shao Y, Du K, Ran L, Fang X, Wang Y (2013). Use of digital gene expression to discriminate gene expression differences in early generations of resynthesized *Brassica napus* and its diploid progenitors. BMC Genomics.

[26] Zhang D, Pan Q, Cui C, Tan C, Ge X, Shao Y, Li Z (2015). Genome-specific differential gene expressions in resynthesized *Brassica* allotetraploids from pair-wise crosses of three cultivated diploids revealed by RNA-seq. Front Plant Science.

[27] Marmagne A, Brabant P, Thiellement H, Alix K (2010). Analysis of gene expression in resynthesized *Brassica napus* allotetraploids: transcriptional changes do not explain differential protein regulation. New Phytol.

[28] Gaeta RT, Yoo S-Y, Pires J, Doerge RW, Chen ZJ, Osborn TC (2009). Analysis of gene expression in resynthesized *Brassica napus* allopolyploids using Arabidopsis 70mer oligo microarrays. PLoS One.

[29] Tan C, Pan Q, Cui C, Xiang Y, Ge X, Li Z (2016). Genome-wide gene/genome dosage imbalance regulates gene expressions in synthetic *Brassica napus* and derivatives (AC, AAC, CCA, CCAA). Front Plant Science..

[30] Zhang D, Pan Q, Tan C, Zhu B, Ge X, Shao Y, Li Z (2016). Genome-wide gene expressions respond differently to A-subgenome origins in *Brassica napus* synthetic hybrids and natural allotetraploid. Front Plant Sci.

[31] Kong F, Mao S, Jiang J, Wang J, Fang X, Wang Y (2011). Proteomic changes in newly synthesized *Brassica napus* allotetraploids and their early generations. Plant Mol Biol Rep.

[32] Albertin W, Balliau T, Brabant P, Chevre A-M, Eber F, Malosse C, Thiellement H (2006). Numerous and rapid nonstochastic modifications of gene products in newly synthesized *Brassica napus* allotetraploids. Genetics.

[33] Zhou R, Moshgabadi N, Adams KL (2011). Extensive changes to alternative splicing patterns following allopolyploidy in natural and resynthesized polyploids. P Natl Acad Sci USA.

[34] Wang Z, Gerstein M, Snyder M (2009). RNA-Seq: a revolutionary tool for transcriptomics. Nat Rev Genet.

[35] Bayer PE, Hurgobin B, Golicz AA, Chan CKK, Yuan Y, Lee H, Renton M, Meng J, Li R, Long Y (2017). Assembly and comparison of two closely related *Brassica napus* genomes. Plant Biotechnol J.

[36] Sun F, Fan G, Hu Q, Zhou Y, Guan M, Tong C, Li J, Du D, Qi C, Jiang L (2017). The high-quality genome of *Brassica napus* cultivar ‘ZS11’reveals the introgression history in semi-winter morphotype. Plant J.

[37] Wang X, Wang H, Wang J, Sun R, Wu J, Liu S, Bai Y, Mun J-H, Bancroft I, Cheng F (2011). The genome of the mesopolyploid crop species *Brassica rapa*. Nat Genet.

[38] Liu S, Liu Y, Yang X, Tong C, Edwards D, Parkin IA, Zhao M, Ma J, Yu J, Huang S (2014). The *Brassica oleracea* genome reveals the asymmetrical evolution of polyploid genomes. Nat Commun.

[39] Jiang J, Rong H, Ran L, Fan H, Kong Y, Wang Y. Creating favorable morphological and yield variations for rapeseed by interspecific crosses between *Brassica rapa* and *B. oleracea*. Plant Breed. 2018; 10.1111/pbr.12602.

[40] Wu J, Zhao Q, Yang Q, Liu H, Li Q, Yi X, Cheng Y, Guo L, Fan C, Zhou Y (2016). Comparative transcriptomic analysis uncovers the complex genetic network for resistance to *Sclerotinia sclerotiorum* in *Brassica napus*. Sci Rep.

[41] Grover C, Gallagher J, Szadkowski E, Yoo M, Flagel L, Wendel J (2012). Homoeolog expression bias and expression level dominance in allopolyploids. New Phytol.

[42] Combes M-C, Cenci A, Baraille H, Bertrand B, Lashermes P (2011). Homeologous gene expression in response to growing temperature in a recent allopolyploid (*Coffea arabica* L.). J Hered.

[43] Rapp RA, Udall JA, Wendel JF (2009). Genomic expression dominance in allopolyploids. BMC Biol.

[44] Hao M, Li A, Shi T, Luo J, Zhang L, Zhang X, Ning S, Yuan Z, Zeng D, Kong X (2017). The abundance of homoeologue transcripts is disrupted by hybridization and is partially restored by genome doubling in synthetic hexaploid wheat. BMC Genomics.

[45] Buggs RJ, Zhang L, Miles N, Tate JA, Gao L, Wei W, Schnable PS, Barbazuk WB, Soltis PS, Soltis DE (2011). Transcriptomic shock generates evolutionary novelty in a newly formed, natural allopolyploid plant. Curr Biol.

[46] Hegarty MJ, Barker GL, Wilson ID, Abbott RJ, Edwards KJ, Hiscock SJ (2006). Transcriptome shock after interspecific hybridization in *Senecio* is ameliorated by genome duplication. Curr Biol.

[47] Akhunova AR, Matniyazov RT, Liang H, Akhunov ED (2010). Homoeolog-specific transcriptional bias in allopolyploid wheat. BMC Genomics.

[48] Powell JJ, Fitzgerald TL, Stiller J, Berkman PJ, Gardiner DM, Manners JM, Henry RJ, Kazan K (2017). The defence-associated transcriptome of hexaploid wheat displays homoeolog expression and induction bias. Plant Biotechnol J.

[49] Flagel L, Udall J, Nettleton D, Wendel J (2008). Duplicate gene expression in allopolyploid *Gossypium* reveals two temporally distinct phases of expression evolution. BMC Biol.

[50] Zhang T, Hu Y, Jiang W, Fang L, Guan X, Chen J, Zhang J, Saski CA, Scheffler BE, Stelly DM (2015). Sequencing of allotetraploid cotton (*Gossypium hirsutum* L. acc. TM-1) provides a resource for fiber improvement. Nat Biotechnol.

[51] Yang J, Liu D, Wang X, Ji C, Cheng F, Liu B, Hu Z, Chen S, Pental D, Ju Y (2016). The genome sequence of allopolyploid *Brassica juncea* and analysis of differential homoeolog gene expression influencing selection. Nat Genet.

[52] Chen ZJ, Pikaard CS (1997). Transcriptional analysis of nucleolar dominance in polyploid plants: biased expression/silencing of progenitor rRNA genes is developmentally regulated in *Brassica*. P Natl Acad Sci USA.

[53] Ge X, Ding L, Li Z (2013). Nucleolar dominance and different genome behaviors in hybrids and allopolyploids. Plant Cell Rep.

[54] Li Z, Wang Y (2017). Cytogenetics and germplasm enrichment in *Brassica* allopolyploids in China. J Integr Agr..

[55] Zhou J, Tan C, Cui C, Ge X, Li Z (2016). Distinct subgenome stabilities in synthesized *Brassica* allohexaploids. Theor Appl Genet.

[56] Patel RK, Jain M (2012). NGS QC toolkit: a toolkit for quality control of next generation sequencing data. PLoS One.

[57] Kim D, Langmead B, Salzberg SL (2015). HISAT: a fast spliced aligner with low memory requirements. Nat Methods.

[58] Trapnell C, Roberts A, Goff L, Pertea G, Kim D, Kelley DR, Pimentel H, Salzberg SL, Rinn JL, Pachter L (2012). Differential gene and transcript expression analysis of RNA-seq experiments with TopHat and cufflinks. Nat Protoc.

[59] Jones P, Binns D, Chang H-Y, Fraser M, Li W, McAnulla C, McWilliam H, Maslen J, Mitchell A, Nuka G (2014). InterProScan 5: genome-scale protein function classification. Bioinformatics.

[60] Conesa A, Götz S, García-Gómez JM, Terol J, Talón M, Robles M (2005). Blast2GO: a universal tool for annotation, visualization and analysis in functional genomics research. Bioinformatics.

[61] Supek F, Bošnjak M, Škunca N, Šmuc T (2011). REVIGO summarizes and visualizes long lists of gene ontology terms. PLoS One.

[62] Langmead B, Salzberg SL (2012). Fast gapped-read alignment with bowtie 2. Nat Methods.

[63] Li H, Handsaker B, Wysoker A, Fennell T, Ruan J, Homer N, Marth G, Abecasis G, Durbin R (2009). The sequence alignment/map format and SAMtools. Bioinformatics.

